# A Green and Practical Magnetic Ionic Liquid-Based Microextraction of DNA Using a Low-Cost 3D-Printed Open-Source Apparatus

**DOI:** 10.1021/acsomega.5c11993

**Published:** 2026-05-17

**Authors:** Luiz C. Ferreira Neto, Mônica Silva Alves, Sofia Aquino Monteiro, Vinícius Flach, Marília Zandoná, Grasiela Agnes, Josias Merib

**Affiliations:** † 117303 Universidade Federal de Ciências da Saúde de Porto Alegre, Porto Alegre, Rio Grande do Sul 90050-170, Brazil; ‡ Programa de Pós-Graduação em Biociências, Universidade Federal de Ciências da Saúde de Porto Alegre, Porto Alegre, Rio Grande do Sul 90050-170, Brazil; § Departamento de Farmacociências, Universidade Federal de Ciências da Saúde de Porto Alegre, Porto Alegre, Rio Grande do Sul 90050-170, Brazil

## Abstract

The analysis of nucleic acids offers numerous possibilities such as identification of varied diseases, determination of specific health conditions, and monitoring of the presence of microorganisms. However, DNA extraction remains a critical step that requires cost-effective, time-saving, and efficient experimental workflows that do not hinder PCR amplification. In this study, a novel high-throughput and cost-effective microextraction strategy for DNA isolation using a lab-made open-source 3D-printed magnetic platform was proposed. This affordable multiwell magnetic platform was built using 3D-printed components coupled with electronic modules, capable of promoting *z*-axis movements using Arduino. In this configuration, 12 magnetic pins were used to hold droplets of the magnetic ionic liquid [P_6,6,6,14_^+^]­[Ni­(hfacac)_3_^–^], enabling multiple extractions. Only 6.5 μL of the MIL was required for each extraction, and the optimized experimental conditions consisted of 600 μL of sample, extraction time of 25 min, and sample pH at 8, while desorption was performed in 300 μL of Tris-HCl (pH 5.5) for 10 min prior to qPCR analysis. The method exhibited satisfactory performance for capturing the BRAF gene with a linear range from 0.005 to 5 pg/μL, with relative recoveries ranging from 92.2 to 117.0%. Intraday precision ranged from 5.0 to 7.3%, interday precision varied from 5.9 to 11.6%, and the limit of quantification (LOQ) was determined as 0.005 pg/μL. The approach was successfully employed to analyze whole-blood samples, demonstrating its applicability to complex biological matrices. The sustainability and applicability assessments resulted in an SPMS score of 7.58 and a BAGI score of 62.5, indicating that the method is both sustainable and practical. Overall, the low-cost open-source device associated with MIL-based SDME offers an efficient, customizable, and accessible alternative for DNA extraction, with potential for further full automation.

## Graphical abstract

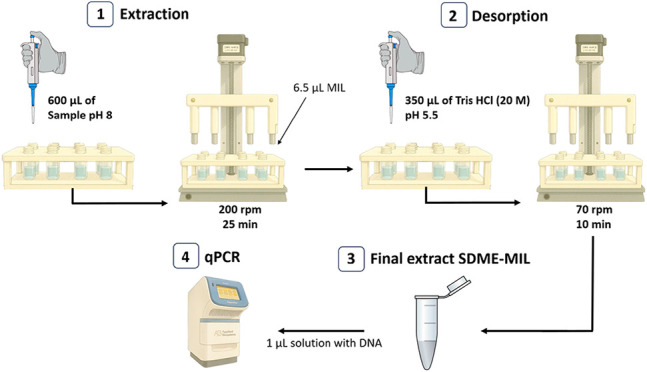

## 1 Introduction

The analysis of nucleic acids permits identification and characterization of pathogens, forensic investigations, as well as the possibility of biomarker detection.^1–3^ Among these analytical approaches, the polymerase chain reaction (PCR) is an important technique that allows the amplification of DNA into billions of copies.^4^ Quantitative PCR (qPCR) is a subtype of the conventional method that further enables precise quantification based on the fluorescence signal associated with the quantification cycle (Cq), which correlates with DNA concentration.^5–7^

Sample preparation for the extraction of biomolecules, such as DNA or RNA, plays a crucial role in ensuring reliable amplification results. This step generally consists of cell lysis, separation of the DNA from other cellular structures, DNA precipitation, and purification. Several DNA extraction methods are available; however, most of them are expensive, laborious, and time-consuming.^8,9^ Traditional liquid–liquid extraction (LLE) using phenol-chloroform requires large volumes of toxic solvents, multiple centrifugation steps, and low sample throughput.^10,11^ Moreover, certain amounts of reagents may leave inhibitory residues that hinder PCR amplification.^12^

Recently, microextraction techniques have gained attention in nucleic acid analysis. Single-drop microextraction (SDME) and dispersive liquid–liquid microextraction (DLLME) offer high extraction efficiency requiring significantly lower amounts of solvent and sample.^13,14^ Importantly, alternative solvents such as magnetic ionic liquids (MILs) have been studied for this purpose. MILs are a subclass of ionic liquids (ILs) that contain metals in their chemical structure while maintaining the tunable features of classical ILs. Therefore, MILs can respond to magnetic fields, which is an interesting aspect to be exploited in sample preparation techniques.^15–20^

Recent studies reported the use of MILs combined with microextraction techniques for DNA extraction in aqueous samples,^16,21^ bacteria,^22,23^ plant lysate,^24^ and blood.^25^ Additionally, works involving the determination of specific short-length fragments of DNA such as cell-free DNA (cfDNA) and circulating tumor DNA (ctDNA) and larger fragments such as genomic DNA (gDNA) have been developed.^26–28^ Despite these advances, most MIL-based extraction workflows still exhibit limited sample throughput and lack of electronic automation.

In 2023, our group examined the extraction of DNA through a mechanical and semiautomated SDME-MIL approach that allowed for the analysis of aqueous and whole-blood samples.^29^ This mechanical apparatus was based on the configuration previously proposed by Mafra et al., employed for the determination of environmental contaminants.^30^ The developed SDME-MIL workflow replaced the traditional SDME approach by the application of a series of rod magnets capable of attracting a drop of a magnetic solvent during the extraction and desorption steps. Despite being capable of processing multiple samples, this experimental procedure was manually controlled, which is a limitation in terms of automation.

Recent progress in open-source instrumentation has enabled the integration of 3D printing and Arduino-based electronic control into analytical workflows, allowing for the design of low-cost and customizable sample preparation devices.^29–34^ However, previously reported automated microextraction systems using SDME and dispersive sorbent microextraction typically process only one sample at a time, which significantly reduces the sample throughput.^35,36^ Importantly, to the best of our knowledge, no electronically controlled, open-source, 3D-printed platform has been reported for DNA extraction using MIL-based SDME.

Therefore, the aim of this study was to propose a novel 3D-printed magnetic platform electronically controlled by an Arduino for the extraction/capture of DNA. In this regard, a straightforward SDME approach using the [P_6,6,6,14_^+^]­[Ni­(hfacac)_3_^–^] MIL as the extraction solvent, with subsequent analysis by real-time PCR, was developed, optimized through multivariate strategies, and validated. This affordable lab-made magnetic platform was produced to permit up to 12 extractions simultaneously, and the analytical results were compared with those obtained in previously reported studies. Moreover, as a proof of concept, the prototype was used to analyze a complex biological sample of whole blood. Finally, sustainability and practicality were assessed using the sample preparation metrics of sustainability (SPMS) and the blue applicability grade index (BAGI), respectively.

## 2 Methods

### 2.1 Materials

In this study, ultrapure water (18.2 MΩ cm) obtained from a Milli-Q water purification system (Millipore, Bedford, MA, USA) was used. Also, ammonium hydroxide (30% v/v), anhydrous ethyl ether (99%), and 1,1,1,5,5,5-hexafluoroacetylacetone (99%) were purchased from Sigma-Aldrich (St. Louis, MO, USA). LC-MS grade acetonitrile was obtained from Merck (Darmstadt, Germany) and nickel­(II) chloride hexahydrate from Êxodo Científica (Sumaré, SP, Brazil). qPCR analyses were conducted with StepOne Plus (Applied Biosystems, Waltham, MA, USA), and polymerase chain reactions were performed using PowerUp SYBR Green Master Mix (Applied Biosystems, Waltham, MA, USA). Amplifications of the BRAF gene were performed using the following primers:

Forward: 5′-CTTCATAATGCTTGCTCTGATAGGA-3′

Reverse: 5′-CAGGGCCAAAAAT TTAATCAGTGA-3′

The specific BRAF fragment (CTTCATAATGCTTGCTCTGATAGGAAAATGAGATCTACTGTTTTCCTTTACTTACTACACCTCAGATATATTTCTTCATGAAGACCTCACAGTAAAAATAGGTGATTTTGGTCTAGCTACAGTGAAATCTCGATGGAGTGGGTCCCATCAGTTTGAACAGTTGTCTGGATCCATTTTGTGGATGGTAAGAATTGAGGCTATTTTTCCACTGATTAAATTTTTGGCCCTG) was used to obtain the calibration curve (Synbio Technologies, NJ, EUA). The 3D components were printed using white and gray filaments of polylactic acid (PLA) obtained from Filamentos 3D Brasil (Novo Hamburgo, RS, Brazil).

### 2.2 Instruments

Each 3D-printed component of the extraction platform was planned and designed using the FreeCAD 0.20.2 software (FreeCAD Project Association) and printed using a Creality CR-5 Pro-H printer (Shenzhen Creality 3D Technology Co, Shenzhen, China). The 3D printing components were fabricated using an infill density of 100% and a layer height of 0.2 mm (200 μm). An Arduino Uno microboard was used for electronic integration, Arduino IDE (Version 2.3.4) was used for software scripting, and a NEMA 17 (Oukeda Motor, model OK42HC34–1504A) stepper motor was employed. After the SMDE-MIL procedure, BRAF gene amplifications were performed using a StepOne Plus (Applied Biosystems, Waltham, MA, USA). Melting curves were obtained using the following temperature program: denaturation at 95 °C for 15 s, followed by annealing at 60 °C for 1 min, and then a gradual increase to 95 °C. The initial process was prepared using a reaction volume of 15 μL consisting of 1.0 μL of DNA template; 5.5 μL of ultrapure water; 7.5 μL of PowerUp SYBR Green Master Mix; and 0.5 μL of each primer (10 μM). The equipment was programmed to start at 50 °C for 2 min, subsequent activation at 95 °C for 2 min, followed by 40 cycles of denaturation at 95 °C for 15 s and annealing at 60 °C for 1 min, resulting in a total reaction time of 2 h.

### 2.3 Synthesis and Characterization of the MIL

The MIL [P_6,6,6,14_^+^]­[Ni­(hfacac)_3_^–^] was synthesized and characterized as proposed by Pierson et al.,^38^ with some minor modifications proposed by Neto et al.^29^ The first step consisted of 10 mmol of ammonium hydroxide and 10 mmol of hexafluoroacetylacetone dissolved in 30 mL of ethanol, and then 3.3 mmol of nickel­(II) chloride hexahydrate was added to the reaction flask (kept under stirring for 12 h). Afterward, 1 mmol of the hfacac ammonium metal salt and 1 mmol of trihexyl­(tetradecyl)­phosphonium chloride were dissolved in 30 mL of methanol. Then, the solvent was removed under reduced pressure, followed by the dissolution of the product in 20 mL of diethyl ether. Afterward, numerous washing steps with deionized water were performed until the aqueous phase no longer produced a precipitate in the presence of AgNO_3_(aq). The characterization of the MIL was carried out by analyzing a solution at a concentration of 1.0 ng/mL in a microTOF-Q III mass spectrometer. The analysis was performed with positive ionization of the ESI, and the parameters used in the mass spectrometer are presented in Table S1 of the Supporting Information. Mass spectrometry data were also included in the Supporting Information (Figure S1).

### 2.4 Experimental Procedure

The electronic extraction platform used in this work was based on a recent study proposed by our research group.^37^ This prototype permits *z*-axis movements controlled by Arduino to allow more independent operations. The Arduino script used in this study is shown in Table S2, as well as the electronic configuration is detailed in Figure S2 of the Supporting Information. Additionally, for the first time, a customized 12-pin extraction blade composed of 3D-printed cylindrical supports capable of accommodating small rod magnets was developed for the application in DNA extraction through SDME-MIL. The extraction and desorption parameters were optimized, and the analytical parameters of merit were obtained. The experimental procedure developed in this study consisted of the following stages:

(i) The extraction step was first optimized in aqueous sample (600 μL) containing 1.0 μg/mL of BRAF gene (229 bp) under stirring (200 rpm), using [P_6,6,6,14_^+^]­[Ni­(hfacac)_3_^–^] as the extraction solvent (6.5 μL). The variables extraction time and sample pH were optimized by a Doehlert design. In this step, 12 magnetic pins were assembled (3 sets containing 4 pins each) to enhance the sample throughput.
(ii) The desorption step was performed under stirring (70 rpm), and Tris-HCl (20 mM) was employed as desorption solvent (300 μL). The variables desorption time and pH of the desorption solution were optimized by a Doehlert design.
(iii) Finally, the desorption solution enriched with DNA was subjected to the qPCR analysis.

### 2.5 Samples Used for Method Applicability

After determining the analytical parameters of merit, whole-blood samples were collected from volunteers of our research group and immediately analyzed in accordance with the ethical guidelines approved by the Ethics Committee of the Federal University of Sciences of Porto Alegre (n° 5.235.870).

### 2.6 Optimization of Experimental Conditions

The parameters that could affect the extraction and desorption steps were optimized by using Doehlert designs. Particularly, related to the extraction step, the extraction time was evaluated at five levels (10, 15, 20, 25, and 30 min), and sample pH was evaluated at three levels (3, 6, and 9). Regarding the desorption step, desorption time was evaluated at five levels (5, 10, 15, 20, and 25 min), and pH of the Tris-HCl solution was evaluated at three levels (4, 6, and 8). The results obtained were processed using the Statistica 8.0 software (Statsoft, Tulsa, OK, USA). The experiments performed in these optimizations are listed in Tables S3 and S4 of the Supporting Information.

### 2.7 Determination of the Analytical Parameters of Merit

The analytical parameters of merit were obtained according to the Guidelines on Bioanalytical Method Validation.^39^ Linear range, linearity, accuracy (relative recoveries), and intra- and interday precision were examined. For the linearity assessment, a calibration curve was performed using ultrapure water enriched with specific concentrations of BRAF gene (229 base pairs) consisting of six concentration points from 0.005 to 5 pg/μL in triplicate. Precision was evaluated at three concentrations, with intraday (*n* = 3) performed on the same day, and interday (*n* = 9) consisting of analysis on three different days. Finally, four whole-blood samples were analyzed to evaluate the applicability of the method.

### 2.8 SPMS and BAGI Metrics

Two metrics were employed to evaluate the method’s performance in terms of sustainability and practicality. The sustainability of the sample preparation workflow was evaluated using the sample preparation metric of sustainability (SPMS).^40^ This metric assigns a score from 0 to 10, considering factors such as sample and solvent consumption, the number of operational steps, energy requirements, and total waste generated. Higher values correspond to more-sustainable procedures.

The Blue Applicability Grade Index (BAGI)^41^ was applied as a complementary evaluation to sustainability metrics and is based on the principles of white analytical chemistry, in which the applicability or practicality of the method is examined. Features including sample throughput, type of analysis, solvent requirements, and automation degree are considered, resulting in a scale from 0 to 100. The result can be visualized as a blue-shaded pictogram in which darker shades correspond to higher applicability.

## 3 Results and Discussion

### 3.1 Production of the Extraction Platform

The 3D-printed platform developed in this study is shown in Figure 1(A–E). The apparatus was designed to accommodate 12 vials simultaneously, and the design considered both the shaker dimensions and the geometry of standard 1.5 mL microtubes. The cylindrical supports were designed according to the diameter of the neodymium rod magnets used to fabricate the platform. Specifically, 6.5 μL of the extraction phase (MIL) was pipetted into the tip of each rod magnet according to the position shown in Figure 1A; afterward, the extraction blade was assembled to the electronic platform, and the magneto-active solvent droplets were immersed into the samples. The 3D-printed base platform permits *z*-axis movement controlled by Arduino, which enables the simultaneous immersion and withdrawal of the MIL droplets from all vials, reducing operator intervention. In our previous study, the device allowed for the simultaneous processing of a larger number of samples; however, that system was semiautomated with manual positioning and operator-dependent control of the extraction/desorption steps.^29^ In contrast, the platform proposed in this work introduces electronically controlled movement using an Arduino-based *z*-axis system, combining a set of 3D-printed supports capable of accommodating a series of rod magnets. This fact can reduce manual labor necessary in the experimental workflow, as well as increase the analysis reproducibility.

**Figure 1:**
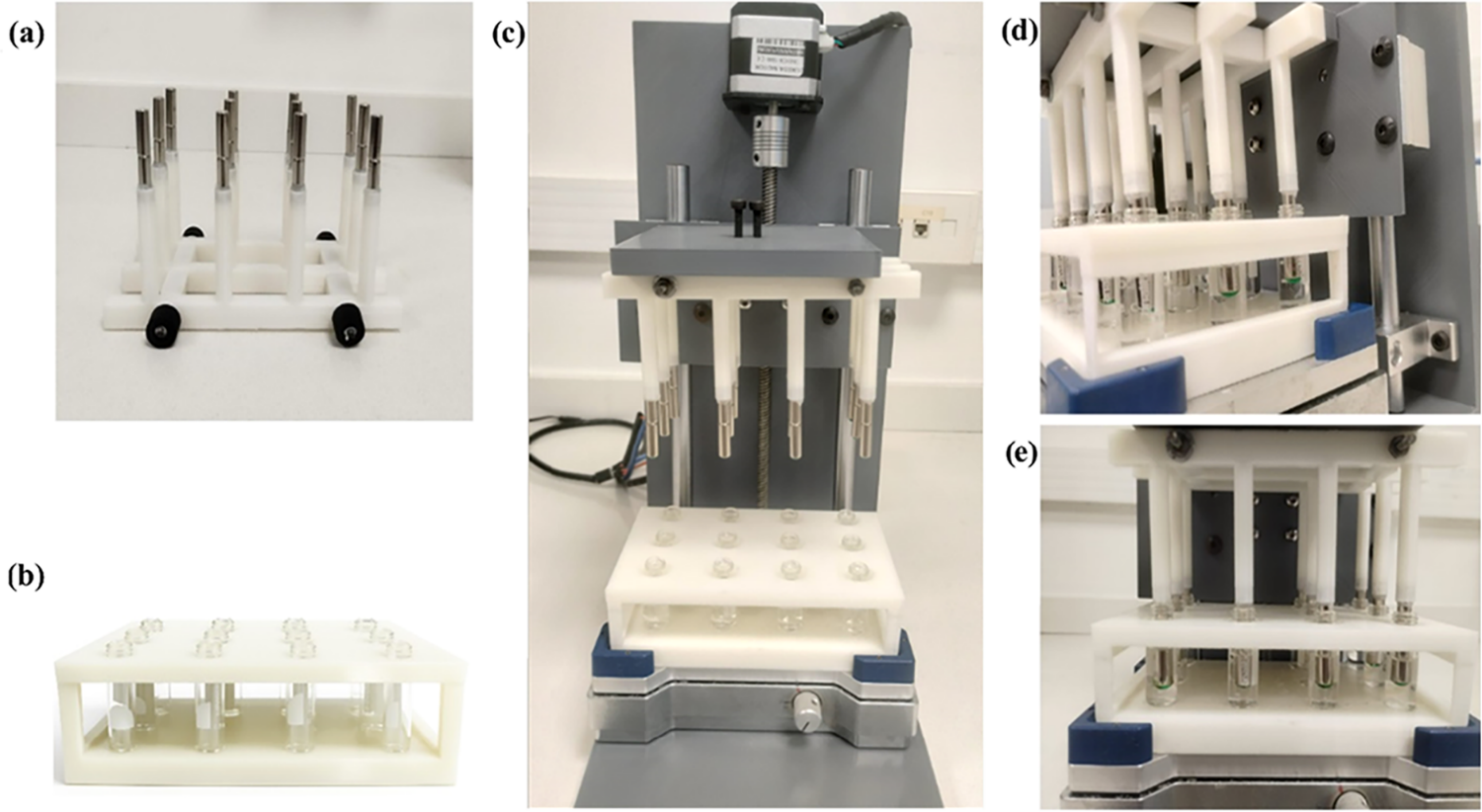
Photographs of the electronic platform developed in this study. (A) Extraction blade composed of a set of 12 pins. (B) 3D-printed base to accommodate the sample vials. (C) Overview of the electronic platform containing magnetic pins for DNA extraction. (D) Lateral visualization of the extraction procedure. (E) Frontal visualization of the extraction procedure.

A more detailed description and dimensions of the 3D-printed components are listed in Figure 2. The base of the platform (Figure 2A) consists of 12 vial supports spaced 31 mm apart horizontally and 28 mm vertically. Each position, with a diameter of 13 mm, was precisely designed to hold the vials securely. The design focused on mechanical stability - with a small hole to fit the vial properly and to permit a complete visualization of the extraction/desorption steps. Each extraction support contains 4 cylindrical pins (42 mm length, 7 mm diameter) with internal 5 mm cavities precisely dimensioned to accommodate neodymium rod magnets (Figure 2B). Three sets of four supports were aligned using 3D-printed spacers (Figure 2C) to ensure that the MIL droplets maintained at the magnet tips are properly positioned inside each vial.

**Figure 2:**
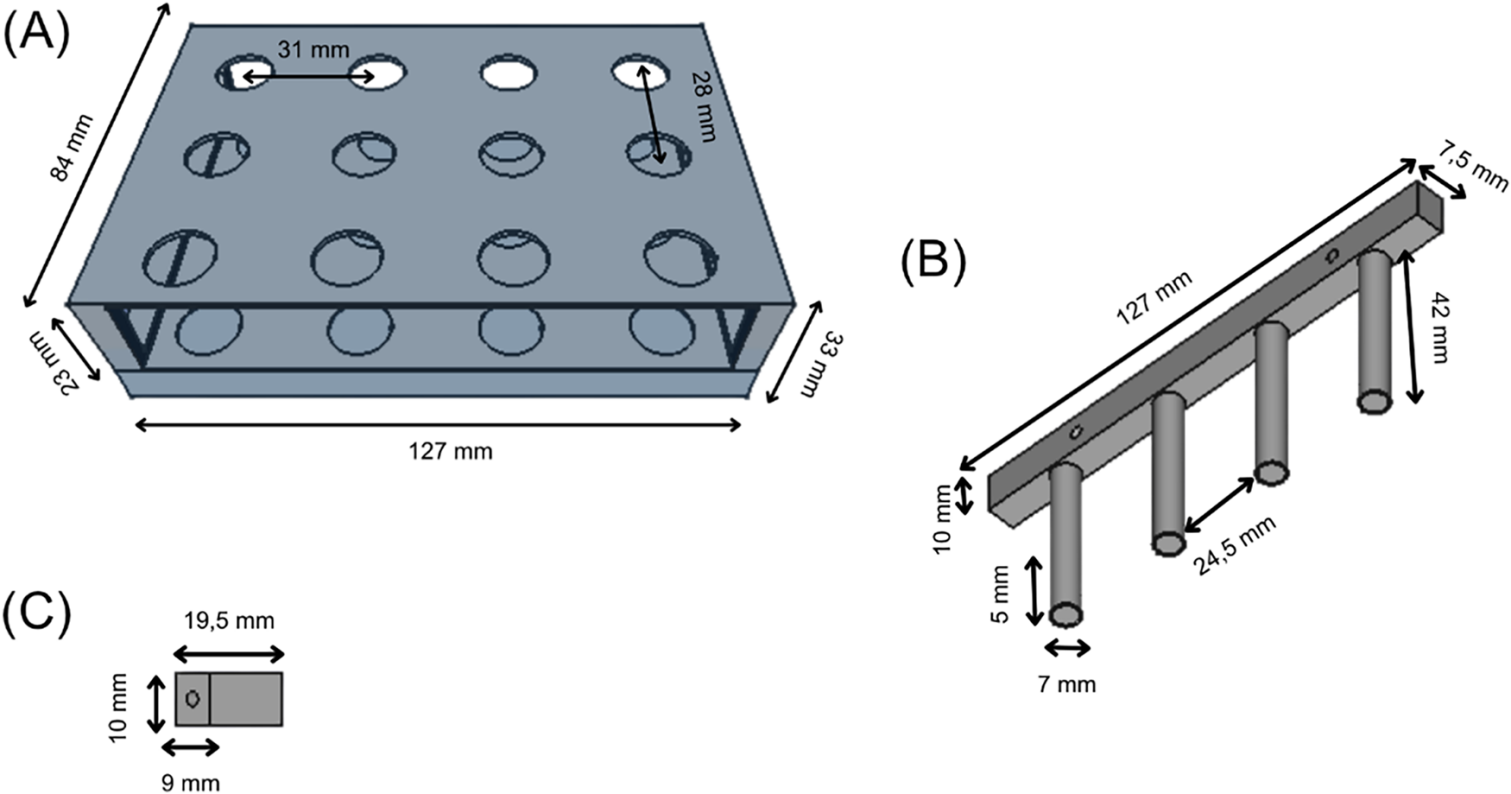
FreeCAD schemes of the platform base and support for rod magnets. (A) Base to accommodate the sample vials. (B) Support for rod magnets. (C) Spacer.

Importantly, there is no contact between the 3D-printed components and the samples during the experimental workflow. Only the rod magnets containing the extraction phase (MIL) were immersed in the samples to permit the DNA extraction. This is particularly important because some polymers can strongly retain DNA, hindering the extraction.^42^ Therefore, the use of glass vials did not exhibit any issue since they were properly washed after each extraction batch.

To the best of our knowledge, no 3D-printed lab-made extraction platform associated with magnetic components has been previously reported for DNA extraction. In contrast to earlier studies relying on manual manipulation or single-sample analysis,^29,35,36^ the integration with an electronic microboard (Arduino) provided a cost-effective alternative associated with high-throughput and straightforward experimental approach. While typical mechanical systems cost around USD 3000 and electronic components approximately USD 5000, the total cost of the proposed sample preparation device was USD 490. The magnetic stirrer was the most expensive component (USD 400), whereas the remaining 3D-printed parts, mechanical, and electronic components were produced or acquired for less than US$ 100. The possibility of handling magnetically active solvents/sorbents using this novel apparatus opens a diverse range of applications in the sample preparation of biomolecules such as DNA.

### 3.2 Optimization

The extraction step was optimized as previously mentioned, and the results are shown in the response surface of Figure 3A. In this case, a coefficient of determination (*r* ^2^) of 0.8966 indicated satisfactory performance of the statistical model. According to Figure 3A, the use of a sample pH adjusted to 8 and an extraction time of 25 min provided the best results. Related to the desorption step, the use of 10 min and a buffer solution adjusted to pH 5.5 resulted in superior performance according to Figure 3B. The statistical model also provided satisfactory adjustment with *r* ^2^ = 0.9399, and no lack of fit was observed. The ANOVA tables for both extraction and desorption steps are shown in Tables S5 and S6 of the Supporting Information.

**Figure 3:**
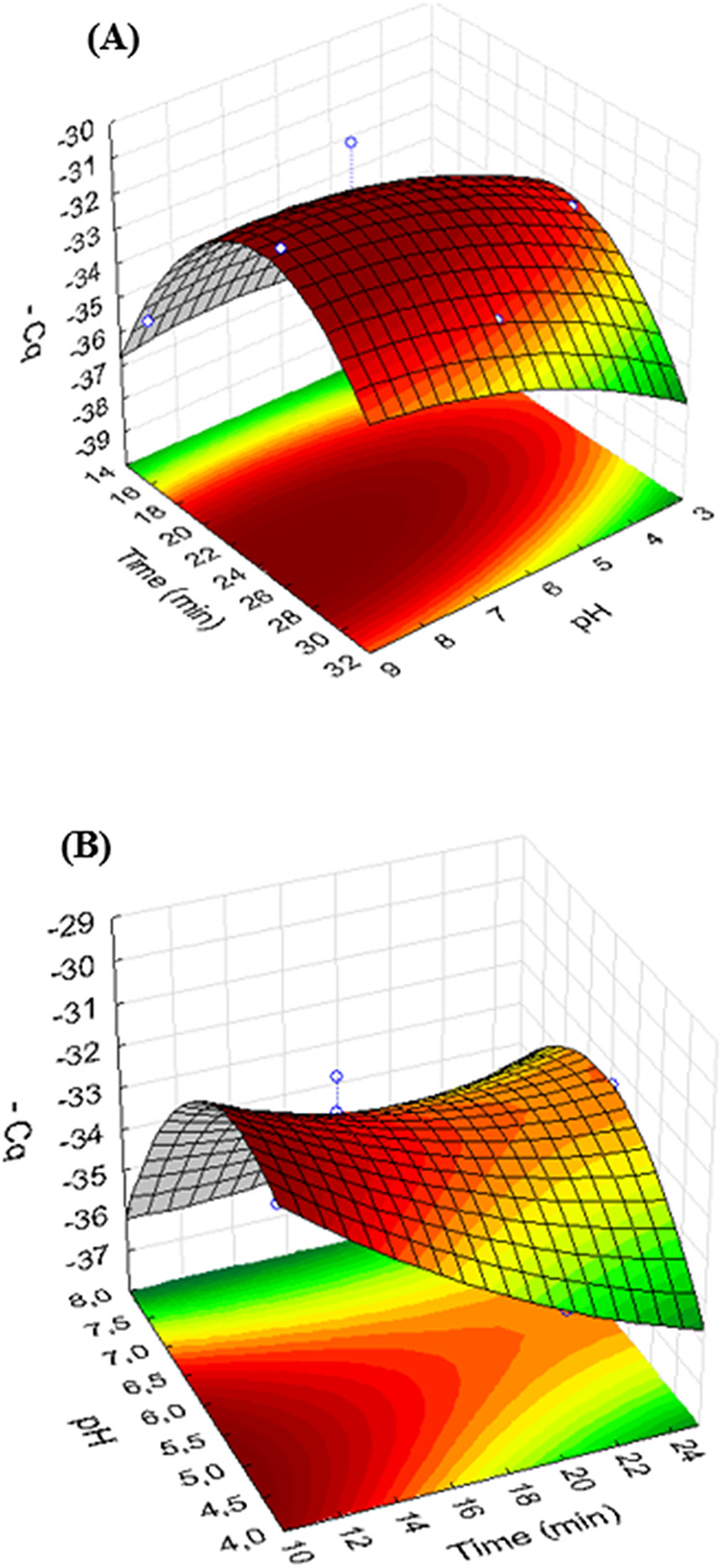
Response surfaces obtained for the optimization of: (A) extraction conditions involving time and sample pH and (B) desorption conditions involving time and pH of the buffer solution.

The extraction and desorption time combined required only 35 min to be completed with minimal intervention. In this case, the use of a potentiometer permits the *z*-axis movements of the platform containing the extraction blade (composed of 12 pins). Therefore, the low-cost electronic platform is simple, easy to control, and customizable according to the application.

Specifically, the pH range for the extraction step is compatible with blood, semen, and saliva matrices.^43^ For urine samples, pH verification and adjustment are recommended since pH can vary from 4.5 to 8. In general, the pH of stool samples and forensic blood samples is commonly below 6.0.^44^ Moreover, DNA extraction from stool samples targeting microbiome analysis should consider the impact of the method on microbiomes.^45^

### 3.3 Experimental Workflow

A general overview of the methodology after optimizing the SDME-MIL workflow is shown in Figure 4. In this optimized procedure, 600 μL of sample adjusted to pH 8 was added to a 1.5 mL vial, under constant stirring (200 rpm), and the extraction was performed with 6.5 μL (pipetted in each tip of the rod magnets) of the MIL of [P_6,6,6,14_^+^]­[Ni­(hfacac)_3_^–^] for 25 min. It is worth mentioning the high mechanical stability of the MIL adhered to the tip of the rod magnet, in contrast to conventional SDME-based procedures in which the solvent drop can be easily lost during extraction. After the extraction step, the DNA-enriched MIL was immersed in a Tris-HCl buffer solution (20 mM and pH 5.5), under constant stirring (70 rpm), for 10 min to desorb the analyte. Finally, 1 μL of this buffer solution with DNA is subjected to qPCR analysis.

**Figure 4:**
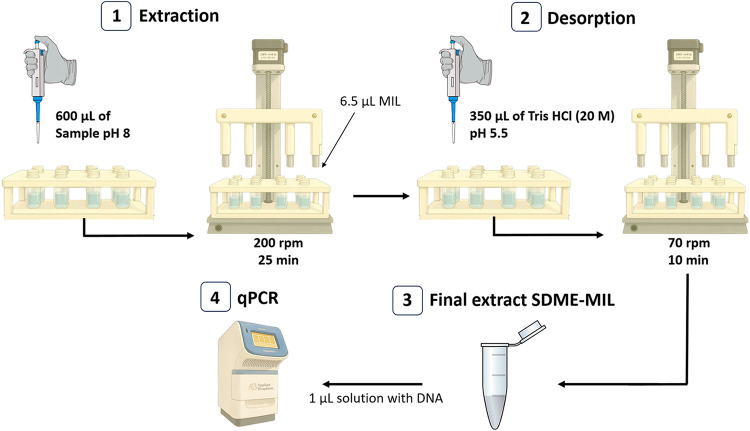
Experimental workflow optimized for the extraction and analysis of DNA.

Two negative controls were included in the extraction experiments to ensure the reliability of the results: (i) extraction performed in the absence of MIL, in which the magnetic rod was introduced into the spiked aqueous sample without the MIL, and (ii) extraction performed with MIL without the presence of the DNA template. Both controls were subjected to the same treatment as the samples and subsequently analyzed by qPCR. None of the controls generated an amplification curve, which indicated no contamination.

As the curve was performed in aqueous samples with a defined fragment, the authors also performed a control in which 0.1 pg/μL of a HBB DNA fragment of 209 bp was added to 0.1 pg/μL of *BRAF* fragment. No significant *C*_t_ value alteration was observed, indicating that the presence of a second fragment with similar length and different sequence composition did not affect amplification performance. In this evaluation, the difference in *C*_t_ value was only 2.5%, which is acceptable for a solvent-based microextraction approach.

### 3.4 Method Validation and Analysis of Real Blood Samples

After the optimization, the analytical parameters of merit were obtained. The calibration curve was performed in triplicate for each concentration, with a linear range from 0.005 to 5 pg/μL. The linear equation was obtained (*y* = −3.4194*x* + 31.27) with a coefficient of determination (*r* ^2^) of 0.9906, indicating satisfactory linearity. This calibration allowed for a calculated amplification efficiency of 96%, considering the slope of the analytical curve, which falls within the accepted range (90 to 110%) for qPCR assays. Intraday precision ranged from 5.0 to 7.3%, and interday precision varied from 5.9 to 11.6%. Accuracy ranged from 92.2 to 117.0%. The results are shown in Table 1 and were considered satisfactory according to the validation guidelines employed.

**Table 1:** Relative Recovery and Precision for Target BRAF Gene in Aqueous Sample

			precision (%)	
target gene	concentration (fg/μL)	relative recovery (%) *n* = 3	intraday (*n* = 3)	interday (*n* = 9)
BRAF	5.0	117.0	5.0	5.9
	100.0	98.5	5.5	11.6
	5000.0	92.2	7.3	8.5

Finally, the electronically controlled SDME-MIL methodology was applied to analyze four whole-blood samples donated by volunteers from our research group. The BRAF gene was detected in all analyzed samples, as shown in Figure 5A. Based on the calibration curve, the concentration of the BRAF gene in whole-blood samples ranged from 12.7 to 21.1 fg/μL. In the other two samples, the BRAF gene was identified; however, the *C*_q_ values did not allow quantification, as they were lower than the studied linear range. The melting curve presented in Figure 5B indicates that all samples were amplified from the same fragment, with no evidence of contamination.

**Figure 5:**
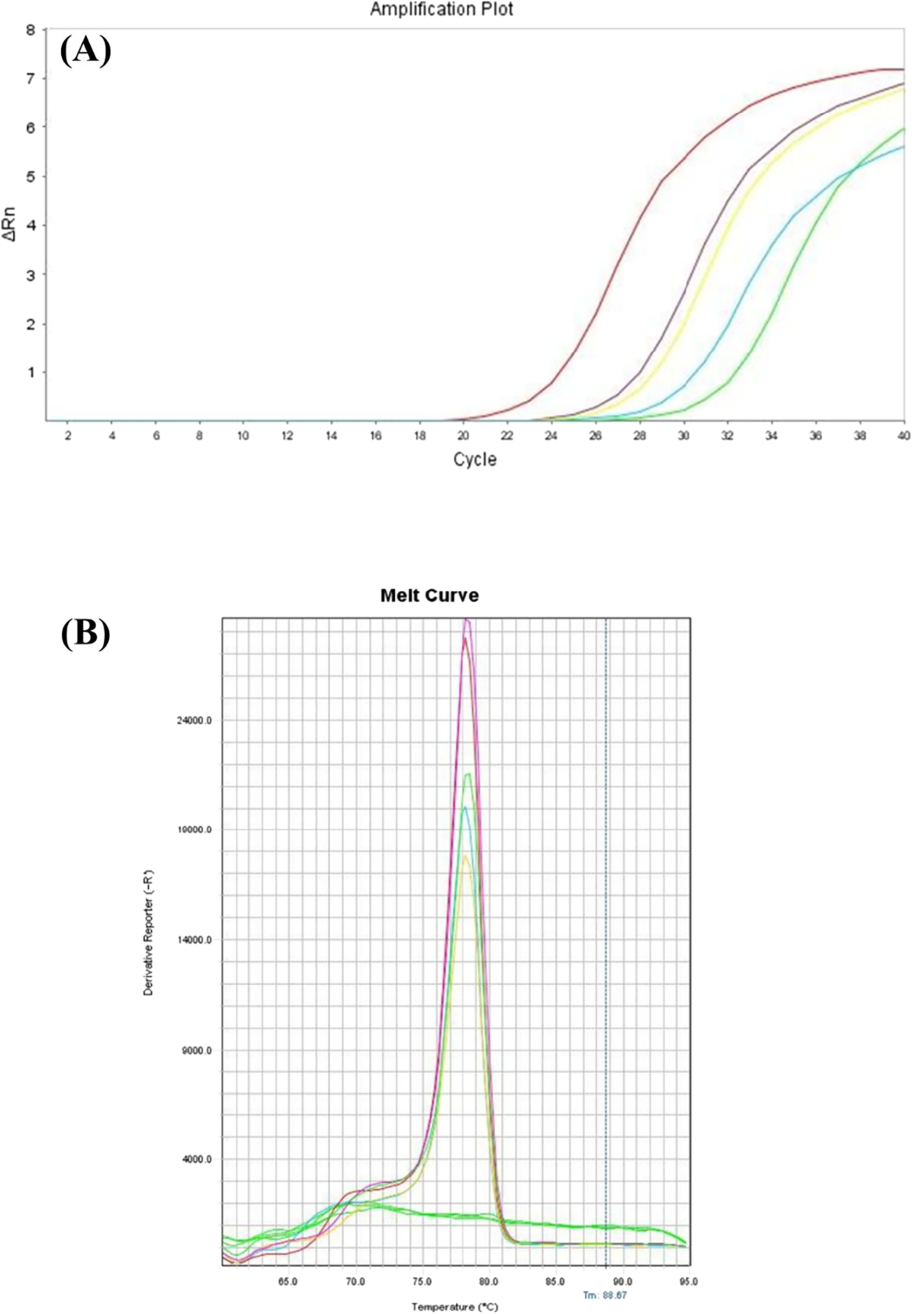
(A) Amplification curves of each sample. The red line corresponds to the positive control. (B) Melting curve from each sample. Green lines at the bottom consist of negative controls.

The analytical features of this methodology were also compared to those of previously reported approaches according to Table 2. This platform presents a low extraction time compared to conventional and some emergent extraction techniques. Classical techniques involving extraction with phenol/chloroform and the rapid method demand a relatively high amount of organic solvents and require several steps such as centrifugation and overnight storage, thus presenting the highest time needed for the extraction.^46^ Moreover, organic solvents often persist after extraction, thus needing additional cleanup before PCR, and are capable of interfering in the PCR reaction. Magnetic beads are an automated method available commercially, but this strategy also requires several washing steps that generate residues, and the instrumentation is costly.^47^ Therefore, the strategy proposed in this study involving 3D printing associated with the electronic microboard is affordable, efficient, and customizable. This platform was able to process 12 samples simultaneously, and the MIL was capable of extracting DNA from complex samples, such as whole blood, without any pretreatment.

**Table 2:** Analytical Features of Sample Preparation Methodologies for DNA Extraction

technique	sample volume (μL)	solvent volume (μL)	time	linear range (pg/μL)	PCR compatibility^a^	additional cleanup	cost per sample^b^	number of samples	refs
Solvent extraction (Phenol/chloroform)	500	3000	2 days		Good	Yes	Low	1	^46^
Rapid method	500	4700	120 min		Moderate	Yes	Low	1	^46^
Magnetic Beads	400		60 min		Excellent	No	High	16	^47^
Silica Columns (QIAamp DNA Blood Mini Kit)	200		40 min		Excellent	No	High	1	^47^
MIL-DLLME	50	2.0	1 min		Excellent	No	Very low	1	^25^
MIL-SDME	1200	6.5	40 min		Excellent	No	Very low	32	^29^
Electronic platform MIL-SDME	600	6.5	35 min	0.005–5	Excellent	No	Low	12	This work

a PCR compatibility was classified according to the reported amplification performance in the original studies and the known efficiency of each approach in removing PCR inhibitors.

b Costs were based on reagent consumption and commercial kit pricing ranges as well as the need for specific devices.

### 3.5 Sustainability and Practicality Assessment

Sustainability evaluation was obtained through SPMS, which is a specific metric related to the sample preparation step.^40^ Parameters such as sample amount, amount of extractant, number of steps, additional steps after extraction, separation, temperature, and total waste were considered. Regarding this methodology, extraction time and nature of the extractant obtained the lowest scores since the MIL is not biodegradable. Overall, this microextraction method exhibited a global score of 7.58, and the pictogram is shown in Figure 6.

**Figure 6:**
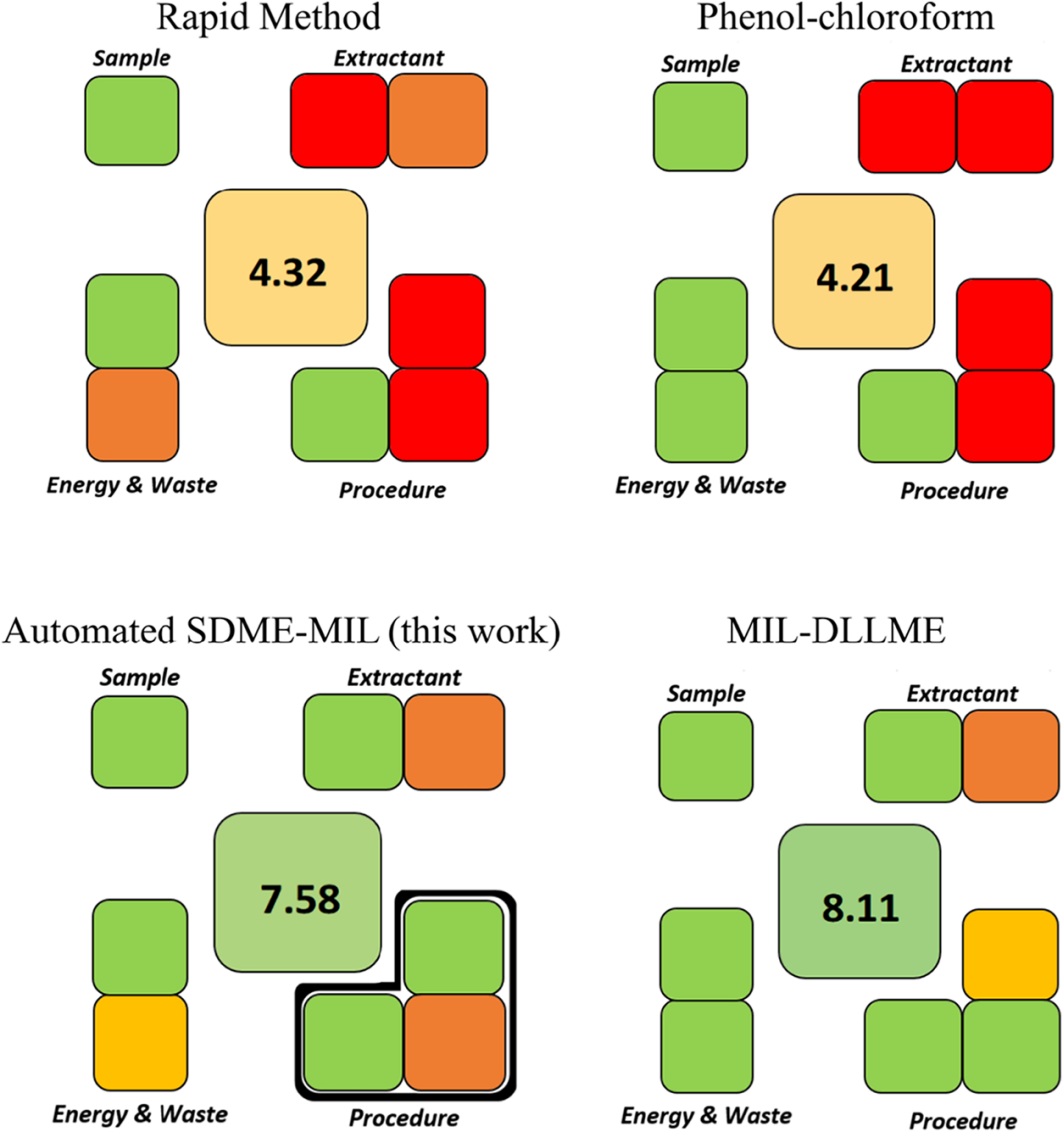
SPMS evaluation for rapid method, phenol-chloroform approach,^46^ MIL-DLLME,^25^ and the proposed method.

According to this metric, the proposed approach can be assigned as “sustainable”. Compared to other approaches for DNA extraction, conventional methods such as phenol-chloroform and rapid extraction protocols exhibited lower SPMS overall scores (4.21), mainly due to the use of toxic solvents and additional sample preparation steps. On the other hand, MIL-based approaches demonstrated significantly improved sustainability performance, with MIL-DLLME reaching a slightly higher score (8.11) due to its lower extraction time and the use of vortex, whereas this work requires a stir plate. Additionally, purification steps using sorbents such as magnetic beads require multiple wash steps and generate a large amount of waste.

The BAGI metric was applied to evaluate the method’s applicability.^41^ According to this metric, the type of analysis was classified as quantitative, since real-time PCR enables the quantification of DNA. Regarding the reagents, MILs are not commercially available, which results in a lower score in the BAGI metric. For the “Reagents and Materials” category, two options were available: “Need to be synthesized with advanced equipment or know-how” and “Need to be synthesized with common instrumentation and in a simple way.” Although the required instrumentation (stirrer, incubator, and rotary evaporator) is common, the synthesis process can take significant time to be completed, thus not meeting the “simple way” criterion. Consequently, this category received the lowest score in the metric. The highlights of this work, according to the BAGI assessment, include its sample throughput (up to 12 samples simultaneously and more than 10 samples per hour). It is also noteworthy that the miniaturized extraction and the degree of automation (“fully automated with novel technological devices, e.g., robotics”) contributed positively to the overall score.

When compared with other DNA extraction methods, the automated SDME-MIL workflow demonstrated higher practicability than conventional approaches such as phenol-chloroform extraction, and performance comparable to magnetic beads-based systems, which achieved the highest BAGI score due to their fully automated commercial platforms. However, these fully automated systems generally exhibit high costs, which can hinder their applicability in some laboratories. Related to the limitation associated with the synthetic procedure of the MIL, this fact can be mitigated by performing the preparation of a solvent batch that can be used for numerous analyses due to the low amount necessary for each extraction. In addition, the use of other qPCR-compatible MILs that exhibit simpler synthesis represents an alternative to increase the practicality of the methodology. Overall, the proposed method achieved a satisfactory score of 62.5 points, which can be considered “practical” according to Manousi et al.^41^ The pictogram obtained in this assessment is shown in Figure 7. The aspects and features evaluated in the SPMS and BAGI metrics are shown in Tables S7 and S8 of the Supporting Information.

**Figure 7:**
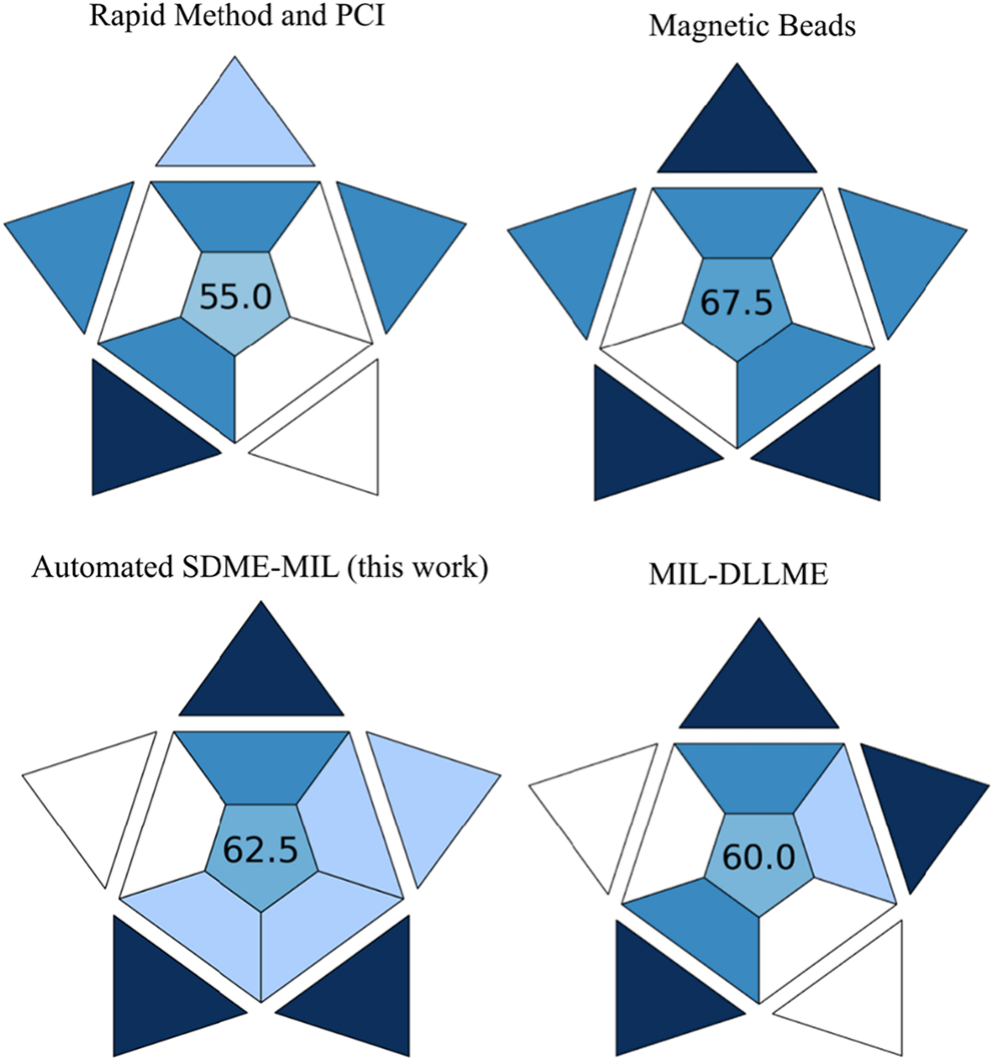
BAGI assessment for rapid method, phenol-chloroform approach,^46^ magnetic beads extraction,^47^ MIL-DLLME,^25^ and the proposed method.

## 4 Conclusion

In this study, for the first time, a magnetic approach was successfully developed for DNA extraction using a lab-made 3D-printed platform coupled to an electronic open-source microboard (Arduino). This affordable and customizable device enabled the simultaneous processing of up to 12 samples within 35 min, including both the extraction and desorption steps. The extraction phase, based on a magnetic ionic liquid, allowed efficient DNA recovery from complex whole-blood samples and was fully compatible with qPCR downstream analysis. Compared to our previously reported semiautomated configuration, this electronically controlled platform provides programmable extraction, representing a transition from manual manipulation to a digitally assisted approach, enabling future automation strategies using accessible open-source technology. The methodology was optimized through multivariate strategies and permitted the quantification of the BRAF gene in real samples. Importantly, features such as low solvent and sample consumption, high throughput, low cost, and the customizable components of this versatile device make this platform a valuable alternative to conventional DNA extraction methodologies. Finally, the approach was evaluated in terms of greenness and practicality, exhibiting satisfactory results for both aspects. On the other hand, the proposed SDME-MIL prototype still exhibits some limitations regarding full automation as certain manual steps are required during sample preparation. Ongoing efforts are focused on developing a fully automated protocol for DNA extraction using an open-source electronic device in the near future.

## Supplementary Material



## Notes

Mass spectrometer parameters and spectra of the MIL, Arduino script, and electronic configuration, matrix of the experiments for the optimization step, ANOVA tables of the optimization step, and aspects and features evaluated in the SPMS and BAGI metrics (PDF)
