# Supplementary material for: A Green and Practical Magnetic Ionic Liquid-Based Microextraction of DNA Using a Low-Cost 3D-Printed Open-Source Apparatus
Source: ACS Omega. 2026 May 17;11(21):30580–90. doi: 10.1021/acsomega.5c11993 (PMC13234795; doi:10.1021/acsomega.5c11993)
Supplement: Supplementary file 1 [file ao5c11993_si_001.pdf]

Supporting Information

A green and practical magnetic ionic liquid-based microextraction of DNA using a low-cost 3D-printed open-source apparatus

Luiz C. Ferreira Neto[1,2], Mônica Silva Alves[1,2], Sofia Aquino Monteiro[1,2], Vinícius Flach[1], Marília Zandoná[1], Grasiela Agnes[1], Josias Merib[1,2,3]*

[1] Universidade Federal de Ciências da Saúde de Porto Alegre, Porto Alegre, RS 90050-170, Brazil;

[2] Programa de Pós-Graduação em Biociências, Universidade Federal de Ciências da Saúde de Porto Alegre, Porto Alegre, RS 90050-170, Brazil;

[3] Departamento de Farmacociências, Universidade Federal de Ciências da Saúde de Porto Alegre, Porto Alegre, RS 90050-170, Brazil.

Corresponding Author

E-mail address: josias@ufcspa.edu.br (J. Merib)

Universidade Federal de Ciências da Saúde de Porto Alegre

245, Sarmento Leite St.

Porto Alegre RS, 90050-170

Phone: +55 51 33038883

Table S1. Instrumental parameters for characterizing the MIL using mass spectrometry.

Parameters of mass spectrometer	Positive ionization	Negative ionization
Acquisition frequency	1 Hz	1 Hz
Capillary voltage	5500 V	4500 V
Drying gas flow	9,0 L/min	8,0 L/min
Drying temperature	250 °C	200 °C
Fragmentation energy	0 eV	0 eV
Ion transfer time	35 μs	120 μs
Monitored mass range	m/z 50–500	m/z 50–800
Nebulization gas flow	3,0 bar	3,0 bar

Table S2. Arduino script used in this study to control the magnetic platform. Obtained with permission from Scheid et al. [37].

```
#include <Stepper.h>
#define potPin 0

const int stepsPerRevolution = 320;
int stepCount = 0;
int valPot = 0;
Stepper myStepper(stepsPerRevolution, 4,5,6,7);

void setup() { }

void loop() {

valPot =  analogRead(potPin);

{
 if (valPot < 470){
 int motorSpeed =map(valPot,500,0,0,100);
 if (motorSpeed > 0) {
   myStepper.setSpeed(motorSpeed);
   myStepper.step(stepsPerRevolution/300);
 }
 }
{
 if (valPot > 530){
 int motorSpeed =map(valPot,515,1024,0,100);
 if (motorSpeed > 0) {
  myStepper.setSpeed(motorSpeed);
  myStepper.step(-stepsPerRevolution/300);
    }
   }
  }
 }
}
```

Table S3. Experiments of the Doehlert design for the optimization of the extraction step.

Experiment	Time (min)	pH
1	10	6
2	15	3
3	15	9
4	20	6
5	20	6
6	20	6
7	25	3
8	25	9
9	30	6

Table S4. Experiments of the Doehlert design for the optimization of the desorption step.

Experiment	Time (min)	pH
1	5	6
2	10	4
3	10	8
4	15	6
5	15	6
6	15	6
7	20	4
8	20	8
9	25	6

Table S5. ANOVA Table from the optimization of the extraction step.

Factor	ANOVA; Var.:Cq; R-sqr=,89666; Adj:,63831 (Spreadsheet1) 2 factors, 1 Blocks, 8 Runs; MS Residual=1,493404 DV: Cq				
	SS	df	MS	F	p
(1)pH (L)	3,01088	1	3,01088	2,016119	0,291476
pH (Q)	0,53740	1	0,53740	0,359849	0,609503
(2)Time (L)	4,09644	1	4,09644	2,743022	0,239521
Time (Q)	11,21828	1	11,21828	7,511886	0,111329
1L by 2L	0,48071	1	0,48071	0,321890	0,627666
Error	2,98681	2	1,49340		
**Total SS**	28,90233	7			

Table S6. ANOVA Table from the optimization of the desorption step.

Factor	ANOVA; Var.:Cq; R-sqr=,93996; Adj:,78987 (Spreadsheet1) 2 factors, 1 Blocks, 8 Runs; MS Residual=,9409778 DV: Cq				
	SS	df	MS	F	p
(1)Time (L)	2,36846	1	2,36846	2,51702	0,253521
Time (Q)	0,42075	1	0,42075	0,44714	0,572542
(2)pH (L)	4,15720	1	4,15720	4,41796	0,170317
pH (Q)	11,70210	1	11,70210	12,43611	0,071852
1L by 2L	5,31687	1	5,31687	5,65036	0,140596
Error	1,88196	2	0,94098		
**Total SS**	31,34646	7			

Table S7. SPMS aspects for the experimental workflow developed in this study.

Criteria group	Criteria	Value	Score
Sample	Sample amount (mL or g)	≤ 10	5
Extractant information	Amount of extractant (mL or g)	≤ 0.1	20
	Nature of extractant	Alternative persistant	6
Procedure information	Number of steps	≤ 2	10
	Extraction time (min)	15 < x ≤ 60	3
	Additional steps after extraction	No additional steps	10
	Samples throughput	Multiple samples	3
Energy consumption	Dispersion/stir	Shaker	1
	Separation	No centrifuge	2
	Temperature	Room temperature	5
Total waste	Waste (mL or g)	≤ 10	10
Reusability of extractant	Reusable	No	1
		**Global score**	**7.58**

Table S8. BAGI aspects for the experimental workflow developed in this study.

Criteria group	Criteria	Value	Score
Analytical determination	Type of analysis	Quantitative	7.5
	Multi- or single-element analysis	Single element	2.5
	Analytical technique	Sophisticated instrumentation (homemade automatic systems)	5
Sample preparation	Simultaneous sample preparation	2-12	5
	Sample preparation	Miniaturized extraction sample preparation	5
Analytical determination and sample preparation steps	Samples per h	>10	10
	Reagents and materials	Need to be synthesized in the lab with advanced equipment or know-how	5
	Preconcentration	No preconcentration required	10
	Degree of automation	Fully automated with novel technology advanced devices (robotics, lab-in-syringe, etc.)	10
	Amount of sample	501-1000 µL bioanalytical samples	5
		**Global score**	**62.5**

Figure S1. Mass spectrometry of the $[P_{6,6,6,14}^{+}][Ni(hfacac)_3^{-}]$ MIL.

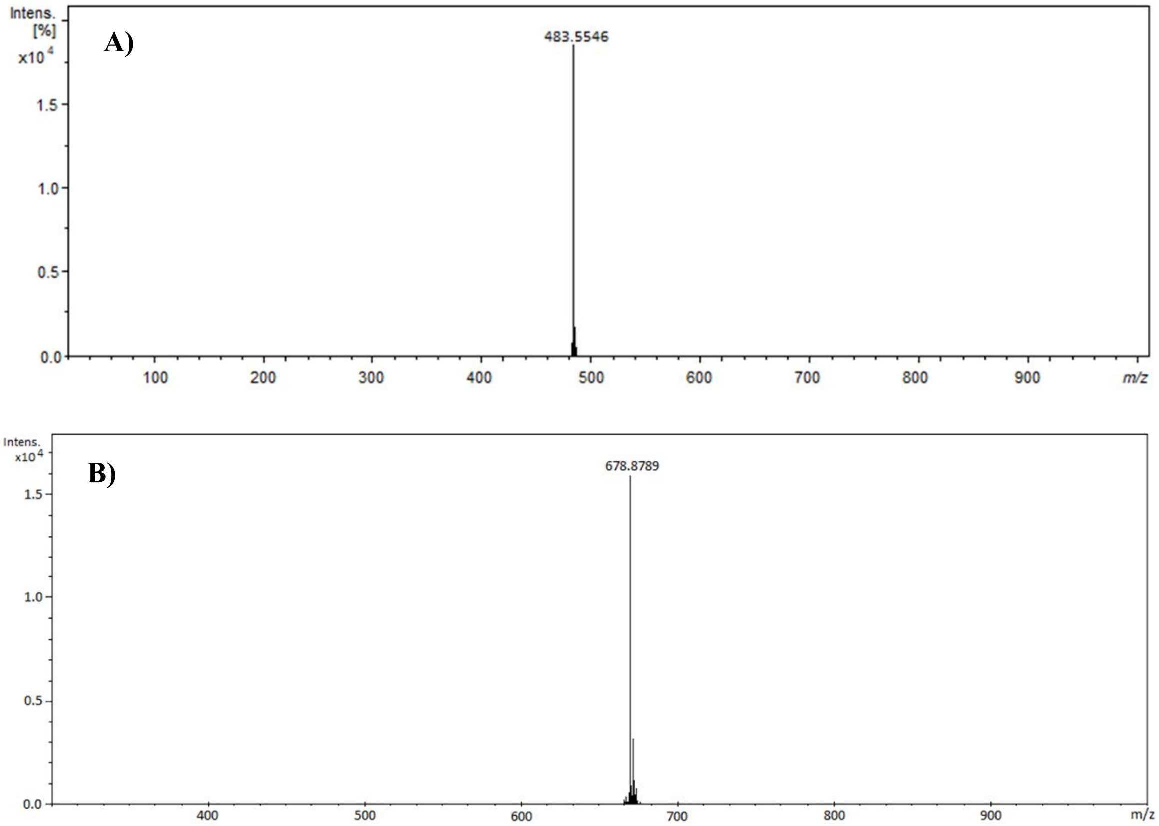

Mass spectrometry of the MIL as previously reported.[29] **A)** cation $[P_{66614}^{+}]$, (ESI+, 1.0 ng mL^{-1}). **B)** anion $[Ni(II)(hfacac)_3^{-}]$ (ESI-, 1.0 ng/mL).

Figure S2. General overview of the components of the sample preparation system. a) scheme of the 3D-printed components designed using FreeCad software; b) mechanical parts which were assembled with the 3D-printed components; c) diagram of the electronic components and connections of the prototype. Adapted with permission from Scheid et al. [37]. Copyright 2025 Elsevier.

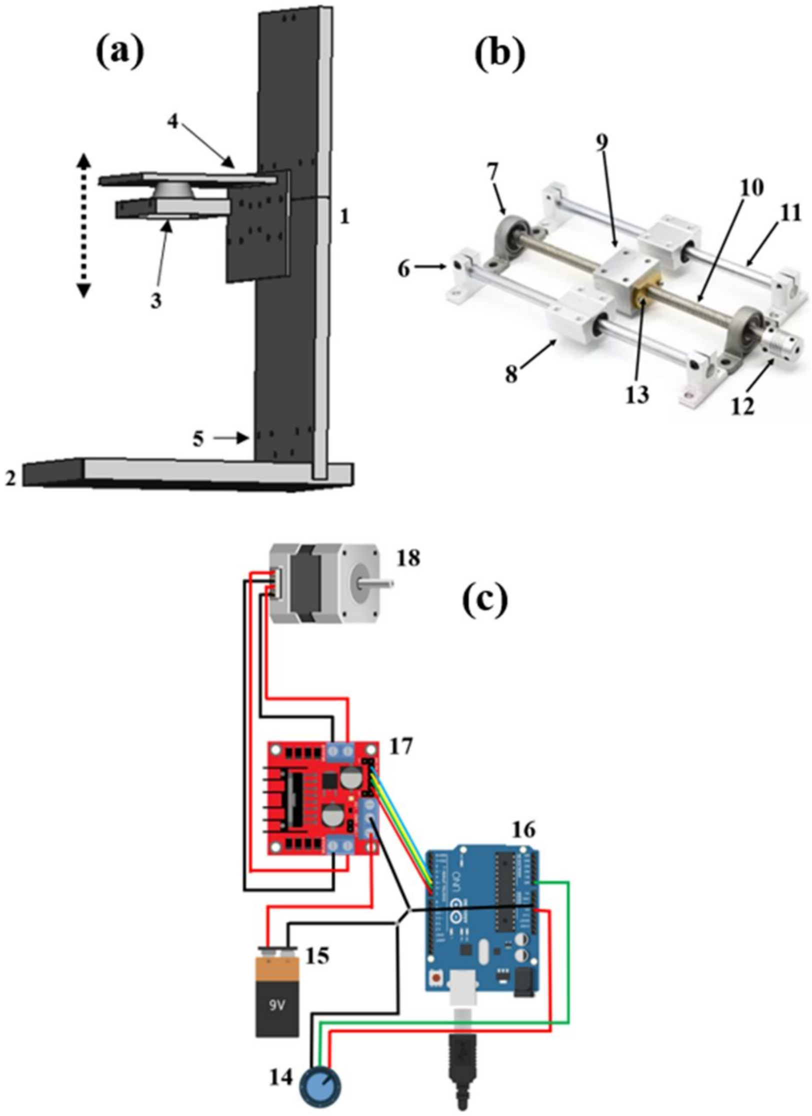

1 – vertical support consisting of two rectangular parts (15 x 13.5 cm; and 15 x 20 cm); **2** – rectangular base designed to accommodate a magnetic stirrer (25 x 18 cm); **3** – rectangular support for holding the set of extraction pins (10.1 x 18.5 cm); **4** – rectangular plate to which the mechanical components were attached to permit up-and-down movements (15 x 7.5 cm); **5** – holes for the attachment of mechanical parts; **6** - stand up shaft support (12 mm); **7** – ball screw 8 mm end support; **8** – pillow block (12 mm shaft); **9** – rectangular aluminum block TR8 (30 mm x 35 mm x 28 mm); **10** – trapezoidal spindle (8 mm x 25 cm); **11** – linear axis shaft (12 mm x 25 cm); **12** – aluminum flexible coupling for stepper motor; **13** – spindle flange; **14** – potentiometer 10k; **15** – 9V battery; **16** – Arduino Uno microcontroller; **17** – L298N motor driver board; **18** – stepper motor NEMA.
